# Supplementary material for: Effects of Monotherapy with Clopidogrel vs. Aspirin on Vascular Function and Hemostatic Measurements in Patients with Coronary Artery Disease: The Prospective, Crossover I-LOVE-MONO Trial
Source: J Clin Med. 2021 Jun 20;10(12):2720. doi: 10.3390/jcm10122720 (PMC8235752; doi:10.3390/jcm10122720)
Supplement: Supplementary file 1 [file jcm-10-02720-s001.zip › jcm-1197815-supplementary.pdf]

Table S1. Antiplatelet regimen, arterial stiffness indices and hemostatic measurement according to endothelial function

	Endothelial dysfunction (RHI < 2.1: n = 50)	Normal endothelial function (RHI ≥ 2.1: n = 28)	*P*
**Antiplatelet regimen**			0.044
DAPT	14 (28.0)	12 (42.9)	
Clopidogrel monotherapy	15 (30.0)	11 (39.3)	
Aspirin monotherapy	21 (42.0)	5 (17.9)	
**Arterial stiffness indices**			
Brachial SBP, mmHg	130.0 ± 20.6	133.9 ± 12.7	0.110
Brachial DBP, mmHg	73.7 ± 12.4	76.0 ± 5.0	0.352
Brachial PP, mmHg	53.2 ± 12.4	57.2 ± 10.5	0.160
Central SBP, mmHg	131.4 ± 22.9	140.4 ± 14.7	0.066
Central DBP, mmHg	73.7 ± 12.4	76.0 ± 5.0	0.352
Central PP, mmHg	57.6 ± 14.0	64.3 ± 13.3	0.043
AI, %	80.6± 12.2	85.2 ± 11.0	0.105
AI@75, %	75.0 ± 12.2	77.9 ± 9.6	0.295
PWV (mean), m/sec	15.2 ± 3.0	16.2 ± 2.1	0.098
Ankle-brachial index (mean)	1.13 ± 0.08	1.13 ± 0.06	0.966
**VerifyNow P2Y12 assay**			
PRU	172 ± 67	140 ± 83	0.065
BASE	209 ± 36	215 ± 38	0.510
**Thromboelastography**			
R, minutes	5.4 ± 1.2	5.6 ± 1.2	0.382
K, minutes	1.8 ± 0.7	1.9 ± 0.8	0.515
Angle, degree	64.5 ± 8.4	60.8 ± 8.4	0.065
$MA_{thrombin}$, mm	61.0 ± 4.3	59.3 ± 6.0	0.141
LY30, %	0.9 ± 1.4	1.9 ± 2.5	0.024

AI, augmentation index; DBP, diastolic blood pressure; PAT, peripheral arterial tonometry; PP, pulse pressure; PRU, P212 reaction unit; PWV, pulse wave velocity; RHI, reactive hyperemia index; SBP, systolic blood pressure.
